# Branched-chain amino acids, arginine, citrulline alleviate central fatigue after 3 simulated matches in taekwondo athletes: a randomized controlled trial

**DOI:** 10.1186/s12970-016-0140-0

**Published:** 2016-07-13

**Authors:** I-Fan Chen, Huey-June Wu, Chung-Yu Chen, Kuei-Ming Chou, Chen-Kang Chang

**Affiliations:** Graduate Institute of Sport Coaching Science, Chinese Culture University, 55, Huagang Rd, Shilin District Taipei, 111 Taiwan; Department of Physical Education, National Taiwan University of Sport, 16, Sec 1, Shuan-Shih Rd, Taichung, 404 Taiwan; Department of Combat Sport, National Taiwan University of Sport, 16, Sec 1, Shuan-Shih Rd, Taichung, 404 Taiwan; Sport Science Research Center, National Taiwan University of Sport, 16, Sec 1, Shuan-Shih Rd, Taichung, 404 Taiwan

**Keywords:** Premotor reaction time, Dual task, Tryptophan, Taekwondo

## Abstract

**Background:**

The decline in cognitive performance has been shown after fatiguing exercise. Branched-chain amino acids (BCAA) have been suggested to alleviate exercise-induced central fatigue. Arginine and citrulline could remove the excess NH_3_ accumulation accompanied with BCAA supplementation by increasing nitric oxide biosynthesis and/or urea cycle. The purpose of this study is to investigate the effect of the combined supplementation of BCAA, arginine, and citrulline on central fatigue after three simulated matches in well-trained taekwondo athletes.

**Methods:**

In a double-blind randomized cross-over design, 12 male taekwondo athletes performed two trials containing three simulated matches each. Each match contained three 2-min rounds of high-intensity intermittent exercise. At the end of the second match, two different supplementations were consumed. In the AA trial, the subjects ingested 0.17 g/kg BCAA, 0.05 g/kg arginine and 0.05 g/kg citrulline, while placebo was consumed in the PL trial. A validated taekwondo-specific reaction test battery was used to measure the cognitive performance after each match.

**Results:**

The premotor reaction time in the three single-task tests and the reaction time in the secondary task in the dual-task test were maintained in the AA trial after three matches, while they were impaired in the PL trial, resulting in significantly better performance in the AA trial. These improvements in the AA trial coincided with significantly lower plasma free tryptophan/BCAA ratio, increased NO_x_ concentrations, and similar NH_3_ concentrations.

**Conclusions:**

This study suggested that the combined supplementation could alleviate the exercise-induced central fatigue in elite athletes.

## Background

The central nervous system plays an important role in development of exercise-induced fatigue [1]. The increased cerebral serotonin (5-hydroxytryptamine) concentration during exercise may be one of the factors responsible for central fatigue. Cerebral serotonin could result in the feeling of lethargy and tiredness, and the loss of central drive and motivation [2]. To support this hypothesis, endurance capacity was significantly decreased by the administration of serotonin agonists, while it was increased by serotonin antagonists in humans and rats [3–5].

In addition to the decreases in muscle output, it has been known that cognitive and skill performance was also impaired as exercise progresses. The studies using functional magnetic resonance imaging have shown that the brain regions involved in high-order motor tasks, such as prefrontal cortex and supplementary motor areas, were affected during fatiguing exercise [6, 7]. As the result, cognitive performance, measured by an auditory choice reaction task and reaction time to visual stimulus, were progressively impaired after exhausting exercise [8, 9]. The performance in a color-word test was also decreased after a 30-km cross-country race [10]. Furthermore, several studies reported impairments in sport-specific skill performance after strenuous exercise [11–13].

Branched-chain amino acids (BCAA), including leucine, isoleucine, and valine, have been suggested to alleviate exercise-induced central fatigue. Plasma BCAA could compete with tryptophan, the precursor for cerebral serotonin synthesis, for the L-system transporter to cross the blood brain barrier [14]. Animal studies have shown that BCAA could increase running time to exhaustion by reducing exercise-induced cerebral synthesis and release of serotonin [15, 16]. However, humans studies have failed to find ergogenic effect of BCAA supplementation [17, 18].

One possible drawback for BCAA supplementation in humans is the excess hyperammonemia due to increased BCAA metabolism during exercise [18–20]. Elevated cerebral uptake and accumulation of NH_3_ would offset the potential benefit of BCAA on central fatigue by alterations of cerebral energy metabolism and neurotransmission, and signaling pathways within the neuron [21]. Arginine has been suggested to reduce exercise-related accumulations of NH_3_ by promoting urea cycle [22] and nitric oxide (NO) biosynthesis [23]. Indeed, the combined supplementation of BCAA and arginine improved intermittent running performance in athletes in 2 consecutive days of simulated handball games by potentially alleviating central fatigue [24]. Citrulline, a NO precursor with high bio-availability [25, 26] and an intermediate in urea cycle [26], could also suppress exercise-induced hyperammonemia [27, 28].

Cognitive function is crucial for success in many sport competitions. However, most aforementioned studies investigating the effect of exhausting exercise on cognitive functions measured the reaction time to various simple stimuli that are dissimilar in sport competitions. Several other studies used pre-determined sport tasks which are closed skills in nature and distinct from the open skills required in many sports. These limitations make it difficult to draw conclusions on the practical application to sport performance in athletes. The present study applied a validated taekwondo-specific reaction battery containing single- and dual-task tests [29] to mimic the cognitive demands in real matches. Three, instead of a single, simulated matches were applied to elicit the physical stress similar to actual competitions. In addition, premotor reaction time (PRT), the difference between the stimulus and the onset of muscle action potentials, was measured to represent the time required for the central nervous system to identify stimuli, process, and transmit signals to the muscles [30]. PRT could be viewed as an indicator for cognitive functions as it excludes the peripheral neuromuscular factors in the reaction process. Therefore, the present study investigated the effect of the combination of BCAA, arginine, and citrulline on the reaction time in a sport-specific setting after high-intensity exercise. Furthermore, the potential mechanisms, including reduced plasma tryptophan/BCAA ratio and enhanced removal of excess NH_3_ by increasing NO biosynthesis and/or urea cycle, were also examined.

## Methods

### Participants

Twelve male taekwondo athletes were recruited from National Taiwan University of Sport, Taichung, Taiwan. All subjects have been participating in taekwondo training for at least 6 years and competed at the national or international level. The subjects have the age of 20.0 ± 0.8 years, the height of 1.77 ± 0.04 m, the weight of 66.9 ± 5.0 kg, the body mass index of 21.29 ± 0.93 kg/m^2^, and VO_2_max of 44.9 ± 6.8 ml/min/kg. The exclusion criteria included cardiovascular disease risks, musculoskeletal injuries, smoking, consumption of protein supplement or under any medication in the past 3 months. The regular training schedule and diet habits were maintained during the study period. The subjects were refrained from all training activity on the day prior to the trial. All subjects gave their written informed consent after the experimental procedure and potential risks were explained. The study protocol was approved by the Research Ethics Committee of China Medical University and Hospital.

### Experimental design

**Fig. 1:**
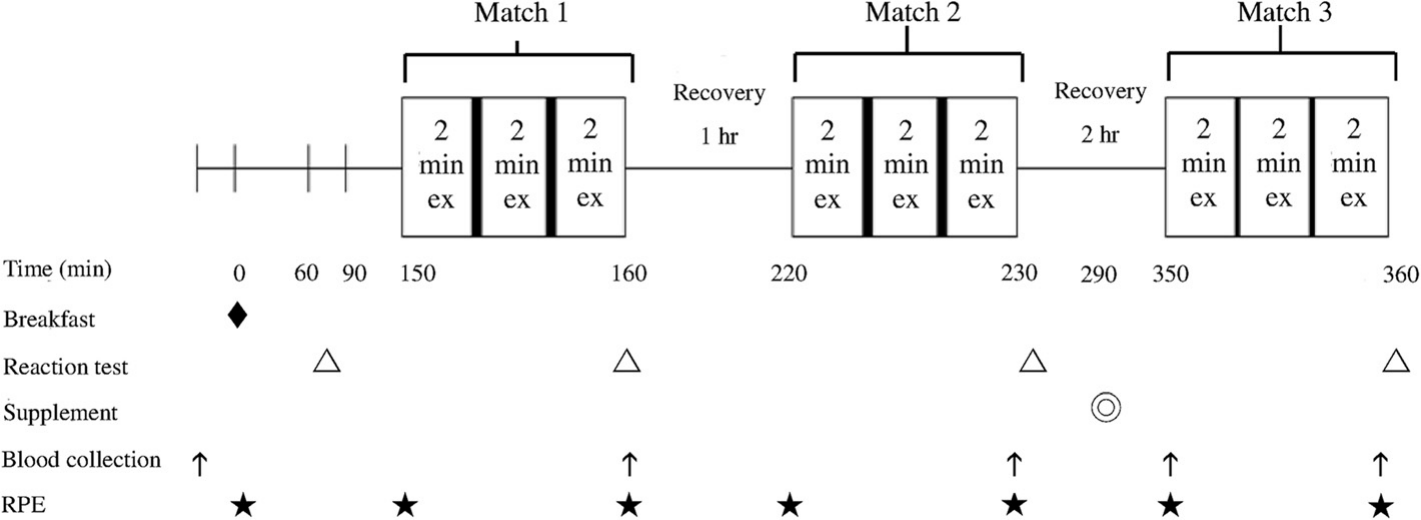
Study design

This study used a double-blind, randomized cross-over design (Fig. 1). Each subject completed amino acids (AA) and placebo (PL) trials in a random order, separated by a wash-out period of at least 7 days. The same food, purchased from local convenience stores, was provided during the 2 days prior to the trials. The meals provided approximately 1800 kcal/day with 54 % energy from carbohydrate, 30 % from fat, and 16 % from protein, according to the manufacturer’s label. The breakfast on the days of trials included white bread 1.2 g/kg, jam 0.1 g/kg, butter 0.l g/kg, and soybean milk 5 ml/kg (6.2 kcal/kg, containing carbohydrate 1.0 g/kg, protein 0.24 g/kg, and fat 0.14 g/kg) [24].

### Experimental procedure

Approximately 1–2 weeks prior to the first trial, cardiopulmonary function was measured. The subjects warmed up on an electrically braked cycle ergometer (Corival, Lode, Groningen, Netherland) at 50 W for 5 min, followed by incremental stages of 25 W every 3 min. The breath-by-breath gas was analyzed (Vmax 29C, Sensormedics, Yorba Linda, CA, USA). Maximal oxygen uptake ($\overset{.}{\mathrm{V}}{\mathrm{O}}_{2 \max }$) was considered to be achieved when the subjects were unable to maintain the workload.

### Supplementation

On the days of the trials, the subjects reported to the laboratory at 0700 after an overnight fast. After collecting venous blood samples as the baseline, the subjects consumed the standardized breakfast. Each trial included 3 simulated matches. After the second match, 2 supplementations were consumed. In the AA trial, the subjects ingested 0.17 g/kg BCAA (leucine: isoleucine: valine = 10:7:3, containing vitamin E 6.67 IU/g BCAA, capsule, General Nutrition Corporation, Pittsburgh, PA, USA), 0.05 g/kg arginine and 0.05 g/kg citrulline (arginine: citrulline = 1:1, tablet, General Nutrition Corporation). In the PL trial, the subjects consumed the identical amount of empty capsule and tablet containing starch (Chung-Yu Biotech Co LTD, Taichung, Taiwan) to the AA trial and 1 capsule of vitamin E (100 IU, General Nutrition Corporation). All supplements were taken with water within 10 min. Our preliminary study has shown that plasma BCAA and arginine concentrations would peak after 1 h of ingestion (data not shown). Therefore, the supplements were consumed 1 h before the third simulated match in this study. In addition, the supplements were given prior to the third match because our pilot study showed that cognitive function started to decline after 2 simulated matches (data not shown).

The subjects were allowed to drink water ad libitum in the first trial, then the timing and amount of consumption were repeated in the following trial. The water consumption was 1133.3 ± 548.6 mL in both trials.

### Simulated match

The simulated match was designed to mimic the high-intensity intermittent nature of actual taekwondo competitions, modified from the previous study [31]. The exercise was performed on a cycle ergometer (894E, Monark, Varberg, Sweden). Each trial contained 3 matches with a 1-hr rest before the second match and a 2-hr rest before the third match. A match included three 2-min rounds with repeated work to rest time of 5 s and 25 s, respectively. A 1-min rest was provided between the rounds. The work to rest ratio of 1 to 5 was determined according to the analysis of international taekwondo matches [32]. During the working period, the load was set at 0.1 kp/kg body weight. The subjects were asked to pedal as fast as possible while the research personnel providing vocal encouragement. The peak and average power of each 5-s sprint was recorded. During within-round and between-round rest periods, the subjects pedaled at 60 rpm without the load.

### Reaction test battery

This taekwondo-specific reaction battery, containing 3 single- and 1 dual-task movements, has been shown to exhibit moderate to high reliability and validity in elite and sub-elite athletes. The intraclass correlation coefficients were 0.439–0.634 in PRT in single-task movements, and 0.692 in reaction time in the secondary task in elite taekwondo athletes [29]. Each of the 4 movements was performed 5 times in a random order. A researcher told the subject which movement was to be performed prior to each task. All subjects were right-handed and performed the kicks with their right leg. Electromyography (EMG) electrodes were attached to left thenar and brachioradialis muscles.

The detailed procedure in this battery can be found elsewhere [29]. Briefly, the subjects stood in a guard position with both heels on a force platform (9260AA6, Kistler, Winterthur, Switzerland) while holding a button on the left hand. The subjects were asked to press the button on the left hand with the thumb as soon as they see a light signal from the top of the head of a dummy, then start the respective movement to attack the dummy. Three sets of single-task movements were used: (A) a roundhouse kick to the rib; (B) a roundhouse kick to the rib, a roundhouse kick to the head, then a reverse roundhouse kick to the head; and (C) a roundhouse kick to the rib, a roundhouse kick to the head, a reverse roundhouse kick to the head, a roundhouse kick to the head, a reverse roundhouse kick to the head, then a roundhouse kick to the head. The subjects can only put the right foot back to the ground after all kicks were performed in the movement. The signals from EMG, force platforms, the button, and the accelerometer (EGAXT3; Measurement Specialties, Hampton, VA, USA) in the dummy were collected through a data acquisition and analysis system (MP150, Biopac Systems, Inc., Goleta, CA, USA).

The dual-task movement D is composed of movement C, the primary task, and a secondary task. While the subjects were carrying out the primary task, research personnel turned on the light signal on the dummy again. The subjects then press the button with their left thumb as soon as they see the second light signal.

In single-task tests, PRT was determined as the time from the beginning of the light signal to the start of EMG signal of the left thenar muscles. The thenar muscles were used because they provided much clearer EMG signal compared to the leg muscles. The EMG signal from leg muscles was noisy due to the unconscious preparation for the kicks before the light, even though the subjects were asked to stand still prior to the stimulus. Motor reaction time was between the start of EMG signal of the left thenar muscles and the right leg leaving the force platform. Movement time was between the right leg leaving the force platform and the appearance of signal from the accelerometer in the dummy.

In the dual-task test, the performance of the secondary task was defined as the time between the beginning of the second light signal and pushing the button. Therefore, the performance of the secondary task involves PRT, motor reaction time, and movement time. EMG signal was not used in measuring the secondary task because it was present throughout the first task. Therefore, it was very difficult to identify the EMG signal that triggered the movement to press the button.

### Blood sample collection

Venous blood samples were collected before breakfast, immediately after each match, and immediately before the start of the third match. At each sampling time, a 16 ml blood sample was collected into a tube containing EDTA. The blood samples were centrifuged at 1500 x g (Eppendorf 5810, Hamburg, Germany) to extract plasma. The aliquoted plasma samples were stored at −70 °C until further analysis.

### Measurement of blood biochemical parameters

Plasma BCAA concentration was measured enzymatically (Biovision, Milpitas, CA, USA). The absorbance at 450 nm was measured with a microplate spectrophotometer (Benchmark Plus, Bio-Rad, Hercules, CA, USA). Plasma free tryptophan concentration was analyzed with a fluorescence assay (Bridge-It, Mediomics, St. Louis, MO, USA). The fluorescence at excitation 485 nm and emission 665 nm was read by a microplate fluorescence reader (Plate Chameleon, Hidex, Turku, Finland). Plasma NOx concentrations were determined using Griess reagent [33] and the absorbance at 450 nm was measured with a microplate spectrophotometer. Plasma concentrations of urea, glucose, lactate, NH_3_, glycerol, and NEFA were measured with an automatic analyzer (Hitachi 7020, Tokyo, Japan) using commercial kits (Randox, Antrim, UK). Plasma concentrations of all parameters were corrected for the changes in plasma volume using hemoglobin concentration and hematocrit in whole blood [34].

### Statistical analysis

All values were expressed as mean ± SD. The results were analyzed by 2-way (trial x time) analysis of variance with repeated measurements. If the time x trial interaction effect is significant, the difference between the 2 trials after the third simulated match was identified by one-way analysis of covariance with the pre-exercise level as the covariant. If the time effect is significant, the differences between each time points within the same trial were determined by post hoc Bonferroni analysis. A *p*-value less than .05 was considered statistically significant.

### Availability of data and materials

The dataset supporting the conclusions of this article is available in ResearchGate (https://www.researchgate.net/publication/303405818_Branched-chain_amino_acids_arginine_citrulline_alleviate_central_fatigue_after_3_simulated_matches_in_taekwondo_athletes_a_randomized_controlled_trial).

## Results

**Fig. 2:**
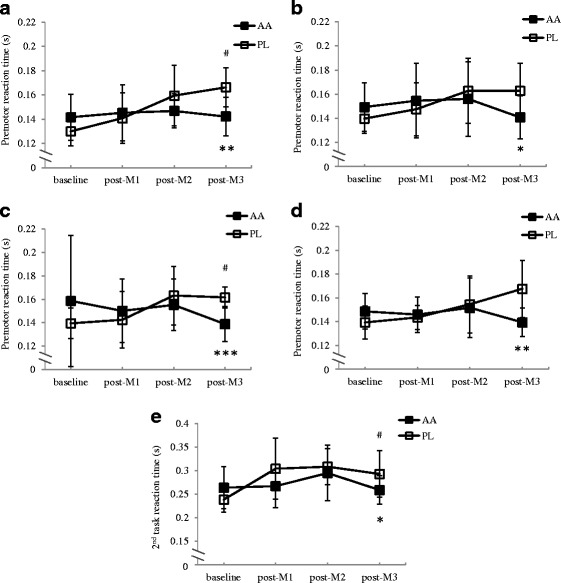
Premotor reaction time in movement A (**a**), movement B (**b**), movement C (**c**), and primary task in movement D (**d**), and reaction time in the secondary task in movement D (**e**) in the taekwondo- specific reaction test battery after each simulated matches in the AA and PL trials^a^. ^a^AA: BCAA, arginine, and citrulline; PL: placebo. (**a**) Main effects: trial: *p* = .156;time: *p* = .001; interaction: *p* = .016; (**b**) Main effects: trial: *p* = .541;time: *p* = .019; interaction: *p* = .025; (**c**) Main effects: trial: *p* = .872;time: *p* = .037; interaction: *p* = .038; (**d**) Main effects: trial: *p* = .113; time: *p* = .152; interaction: *p* = .015; (**e**) Main effects: trial: *p* = .123; time: *p* = .036; interaction: *p* = .018; ^*^ *p* < .05, ^**^ *p* < .01, ^***^ *p* < .001, significantly different between the 2 trials. ^#^Significantly different from the baseline in the PL trial (*p* < .05)

PRT in the movement A in the AA and PL trials is shown in Fig. 2a. There were significant time and trial x time effects. PRT after the third match in the AA trial (0.142 ± 0.016 s) was significantly faster than that in the PL trial (0.166 ± 0.017 s; *F* = 13.03, η^2^ = 0.383, 95 % confidence interval (CI): −0.041, −0.011, *p* = .002). In movement B, PRT after the third match in the AA trial (0.141 ± 0.019 s) was also significantly faster than that in the PL trial (0.163 ± 0.024 s; *F* = 6.61, η^2^ = 0.239, 95 % CI: −0.043, −0.005, *p* = .018) (Fig. 2b). The results were alike in the most complicated movement C (Fig. 2c). In the PL trial, PRT after the third match was significantly deteriorated compared to the baseline level, while it was maintained in the AA trial, resulting in significantly faster PRT in the AA trial (AA trial: 0.139 ± 0.016 s; PL trial: 0.162 ± 0.010 s; *F* = 18.15, η^2^ = 0.464, 95 % CI: −0.035, −0.012, *p* < .001). PRT in the primary task in movement D, which is identical to movement C, exhibited the similar trend (Fig. 2d). The AA trial showed significantly faster PRT after the third match than that in the PL trial (AA trial: 0.139 ± 0.013 s; PL trial: 0.168 ± 0.025 s; *F* = 10.67, η^2^ = 0.337, 95 % CI: −0.047, −0.010, *p* = .004). There was significant time and trial x time interaction effect in reaction time in the secondary task in movement D (Fig. 2e). In the PL trial it was significantly slower after the third simulated match, compared to the baseline level, while it was maintained in the AA trial, resulting in better performance in the AA trial (AA trial: 0.259 ± 0.031 s; PL trial: 0.293 ± 0.051 s; *F* = 5.46, η^2^ = 0.206, 95 % CI: −0.079, −0.005, *p* = .029). There was no significant effect in motor reaction time or movement time in any movement (Table 1).

**Table 1:** Motor reaction time and movement time in the taekwondo-specific reaction test battery after each simulated matches in the AA and PL trials

	Movement^a^	Trial^b^	Baseline (s)	post-M1 (s)	post-M2 (s)	post-M3 (s)
Motor reaction time	A	AA	0.295 ± 0.057	0.267 ± 0.055	0.270 ± 0.050	0.266 ± 0.033
		PL	0.285 ± 0.040	0.265 ± 0.044	0.258 ± 0.048	0.270 ± 0.041
	B	AA	0.313 ± 0.079	0.307 ± 0.069	0.269 ± 0.044	0.299 ± 0.064
		PL	0.314 ± 0.043	0.305 ± 0.054	0.292 ± 0.070	0.303 ± 0.056
	C	AA	0.342 ± 0.066	0.303 ± 0.067	0.287 ± 0.043	0.307 ± 0.038
		PL	0.314 ± 0.055	0.305 ± 0.051	0.304 ± 0.051	0.303 ± 0.055
	D	AA	0.356 ± 0.071	0.304 ± 0.067	0.277 ± 0.047	0.300 ± 0.049
		PL	0.319 ± 0.039	0.307 ± 0.043	0.300 ± 0.071	0.301 ± 0.052
Movement time	A	AA	0.269 ± 0.043	0.250 ± 0.033	0.246 ± 0.030	0.258 ± 0.042
		PL	0.263 ± 0.033	0.260 ± 0.042	0.273 ± 0.039	0.262 ± 0.040
	B	AA	0.278 ± 0.041	0.266 ± 0.035	0.262 ± 0.032	0.263 ± 0.030
		PL	0.279 ± 0.033	0.294 ± 0.071	0.275 ± 0.033	0.272 ± 0.032
	C	AA	0.270 ± 0.050	0.269 ± 0.037	0.264 ± 0.028	0.243 ± 0.066
		PL	0.282 ± 0.036	0.277 ± 0.044	0.276 ± 0.032	0.281 ± 0.035
	D	AA	0.272 ± 0.038	0.272 ± 0.030	0.260 ± 0.032	0.269 ± 0.032
		PL	0.267 ± 0.069	0.274 ± 0.051	0.280 ± 0.034	0.278 ± 0.041

^a^Movement A: a roundhouse kick to the rib; Movement B: a roundhouse kick to the rib, a roundhouse kick to the head, then a reverse roundhouse kick to the head; Movement C: a roundhouse kick to the rib, a roundhouse kick to the head, a reverse roundhouse kick to the head, a roundhouse kick to the head, a reverse roundhouse kick to the head, then a roundhouse kick to the head; Movement D: the primary task in a dual-task setting, same as Movement C. ^b^ *AA* BCAA, arginine, and citruline, *PL* placebo

**Fig. 3:**
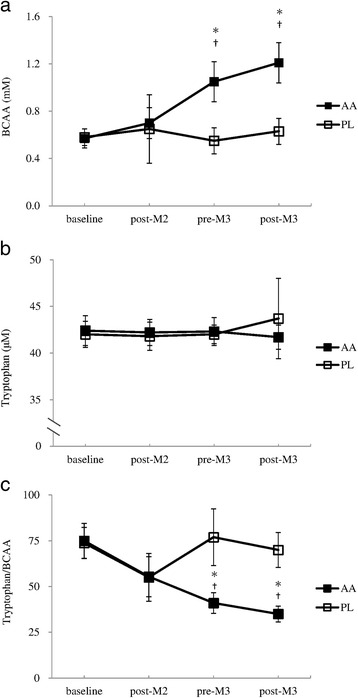
Plasma BCAA (**a**) and tryptophan (**b**) concentrations and tryptophan/BCAA ratio (**c**) in the AA and PL trials^a^. ^a^AA: BCAA, arginine, and citrulline; PL: placebo. (**a**) Main effects: trial: *p* < .001; time: *p* < .001; interaction: *p* < .001; (**b**) Main effects: trial: *p* = .252;time: *p* = .050; interaction: *p* = .196; (**c**) Main effects: trial: *p* < .001;time: *p* < .001; interaction: *p* < .001; ^*^Significantly different between the 2 trials (*p* < .001). ^†^ Significantly different from the baseline in the AA trial (*p* < .05)

The supplementation at 1 h prior to the third simulated match resulted in significantly higher plasma BCAA concentrations before and after that match in the AA trial, compared to those in the PL trial (*F* = 74.78, η^2^ = 0.781, 95 % CI: 0.383, 0.626, *p* < .001; *F* = 114.22, η^2^ = 0.845, 95 % CI: 0.466, 0.692, *p* < .001; respectively, Fig. 3a). Plasma free tryptophan concentrations did not show any change in either trials (Fig. 3b). The significantly elevated plasma BCAA concentration before and after the third simulated match in the AA trial resulted in the significantly lower free tryptophan/BCAA ratio than the PL trial (*F* = 62.74, η^2^ = 0.749, 95 % CI: −49.278, −28.783, *p* < .001; *F* = 126.36, η^2^ = 0.857, 95 % CI: −41.763, −28.723, *p* < .001; respectively, Fig. 3c).

**Fig. 4:**
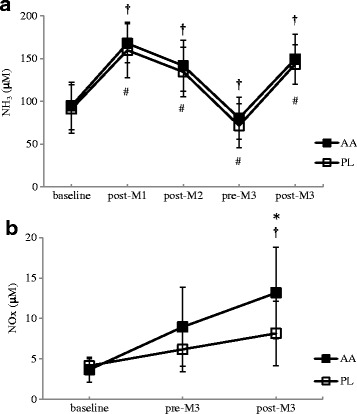
Plasma NH_3_ (**a**) and NO_x_ (**b**) concentrations in the AA and PL trials^a^. ^a^AA: BCAA, arginine, and citrulline; PL: placebo. (**a**) Main effects: trial: *p* = .289;time: *p* < .001; interaction: *p* = .958; (**b**) Main effects: trial: *p* = .252; time: *p* = .050; interaction: *p* = .196. ^*^Significantly different between the 2 trials (*p* < .05). ^†^Significantly different from the baseline in the AA trial (*p* < .05). ^#^Significantly different from the baseline in the PL trial (*p* < .05)

The simulated matches significantly increased plasma NH_3_ concentrations by the similar magnitude in both trials (Fig. 4a). There were not significant differences between the 2 trials. The AA trial showed significantly higher NO_x_ concentrations after the third simulated match (AA trial: 13.2 ± 6.0 μM; PL trial: 8.1 ± 4.3 μM, *F* = 4.705, η^2^ = 0.266, 95 % CI: 0.023, 11.286, *p* = .049; Fig. 4b). The plasma concentrations of urea, glucose, lactate, glycerol, and NEFA did not show any difference between the 2 trials (Table 2).

**Table 2:** Plasma biochemical parameters and ratings of perceived exertions in the AA and PL trials

	Trial^a^	Baseline	post-M1	post-M2	pre-M2	post-M3
Urea (mM)^1^	AA	4.61 ± 1.72	8.20 ± 3.65	12.70 ± 4.50*	13.25 ± 4.10*	17.56 ± 5.30*
	PL	4.55 ± 1.90	7.36 ± 2.30	12.95 ± 5.00*	9.07 ± 4.00	16.92 ± 5.10*
Lactate (mM)^2^	AA	2.95 ± 0.77	13.38 ± 4.17*	12.78 ± 3.77	2.22 ± 0.53	11.93 ± 2.90
	PL	3.19 ± 0.75	13.37 ± 2.96*	12.24 ± 3.45	2.36 ± 0.55	12.57 ± 2.27
Glucose (mM)^3^	AA	5.26 ± 0.28	4.39 ± 0.44*	4.97 ± 0.50	5.37 ± 0.39	5.12 ± 0.50
	PL	5.25 ± 0.44	4.20 ± 0.27*	4.89 ± 0.39	5.36 ± 0.22	5.28 ± 0.39
Glycerol (μM)^4^	AA	27.75 ± 10.37	49.38 ± 22.00	76.48 ± 27.14*	79.81 ± 24.91*	105.78 ± 31.76*
	PL	27.42 ± 11.39	44.34 ± 13.96	78.00 ± 30.22*	54.65 ± 24.31	101.91 ± 30.93*
NEFA (mM)^b,5^	AA	0.33 ± 0.17	0.21 ± 0.08	0.34 ± 0.15	0.38 ± 0.12	0.47 ± 0.20
	PL	0.36 ± 0.16	0.21 ± 0.06	0.38 ± 0.10	0.58 ± 0.24	0.55 ± 0.19
RPE^c,6^	AA	9.3 ± 2.0	16.0 ± 2.0*	15.9 ± 2.0*	-	15.2 ± 3.0*
	PL	9.3 ± 2.0	12.8 ± 2.0*	13.6 ± 2.0*	-	14.6 ± 2.0*

^a^ *AA* BCAA, arginine, and citruline, *PL* placebo. ^b^ *NEFA* non-esterified fatty acid. ^c^ *RPE* ratings of perceived exertion. *Significantly different from baseline in the same trial (*p* < .05)

^1^Main effects: trial: *p* = .329; time: *p* < .001; interaction: *p* = .167

^2^Main effects: trial: *p* = .325; time: *p* < .001; interaction: *p* = .165

^3^Main effects: trial: *p* = .816; time: *p* < .001; interaction: *p* = .520

^4^Main effects: trial: *p* = .325; time: *p* < .001; interaction: *p* = .165

^5^Main effects: trial: *p* = .117; time: *p* < .001; interaction: *p* = .213

^6^Main effects: trial: *p* = .886; time: *p* = .003; interaction: *p* = .718

The average power output in each simulated match was not significantly different between the 2 trials (AA trial: 10.76 ± 1.04, 11.03 ± 0.77, 10.98 ± 0.79 W/kg; PL trial: 11.00 ± 0.46, 10.93 ± 0.68, 10.95 ± 0.58 W/kg, match 1, 2, and 3, respectively). Each simulated matches significantly increased RPE scores by the similar extent, with no significant trial or trial x time interaction effect.

## Discussion

To our knowledge, this is the first study revealing that the combined supplementation of BCAA, arginine, and citrulline could prevent the exercise-induced central fatigue in a sport-specific setting in athletes. After the supplementation, the subjects in the AA trial showed significantly faster PRT in the 3 single-task movements, as well as the reaction time in the secondary task in the dual-task test, compared to those in the PL trial. These improvements coincided with a significantly lower tryptophan/BCAA ratio in the AA trial. Furthermore, the supplementation did not lead to additional NH_3_ accumulation, possibly mediated by an increased NO production from arginine and citrulline.

Previous studies have reported that BCAA supplementation could maintain cognitive functions [10] and the performance in reactive motor skills [35], while reducing the feeling of fatigue [17] during strenuous exercise. However, the accompanied excess NH_3_ accumulation could offset the effect of BCAA in most human studies [18–20]. The addition of arginine and citrulline to BCAA in this study resulted in similar plasma NH_3_ levels between the AA and PL trials. An increased NO production and possibly vasodilation in the AA trial may help to remove the excess NH_3_ produced from the elevated BCAA metabolism. It has been reported that the supplementation of BCAA and arginine after exhaustive exercise could reduce the feeling of fatigue during recovery [36]. The combined supplementation of BCAA and arginine also significantly improved performance in high-intensity intermittent sprints on the second day of 2 consecutive days of exercise by alleviating central fatigue [31]. By incorporating citrulline, a more potent NO precursor than arginine [37], the excess hyperammonemia previously seen after BCAA supplementation was prevented in the present study. Although the lack of a BCAA-only trial in this study precludes the direct conclusion that arginine and citrulline alleviate excess hyperammonemia, the alleviation of central fatigue by this combined supplementation regime is still prominent.

During prolonged exercise, brain regions involved in high-order motor tasks, sensory processing, and corticomotor drive are activated in order to maintain the muscular performance [6, 7, 38]. The progressively increased cerebral processing demand to maintain muscular output could lead to less brain resource that can be allocated to cognitive function. In the PL trial, significant deteriorations in PRT were apparent in all movements after the third simulated match, indicating that the accumulated physical demands resulted in central fatigue. Similarly, it has been reported that exhausting exercise could impair the reaction time in response to different types of stimuli [8, 9]. The sport-specific skill performance was also significantly decreased after exhausting exercise [11–13]. On the other hand, in the AA trial these cognitive performances were maintained and significantly better than those in the PL trial. A significantly lower plasma tryptophan/BCAA ratio, hence reduced cerebral serotonin synthesis [15], could be one of the mechanisms that are responsible for the alleviation of central fatigue.

This study utilized a validated dual-task protocol [29] to further identify the development of central fatigue after exercise and supplementations. The dual-task protocols carry better validity, compared to the single-task, when the primary tasks are very familiar to the participants [29]. It has been hypothesized that human brain has a fixed capacity of central processing. During dual-task situations the majority of the capacity would be distributed to the primary task. As the exercise progresses, the increased demand for the central resource for the primary task may lead to the impaired performance in the secondary task [39]. In the present study, the AA trial showed a significantly faster RT in the secondary task after the third simulated match. This result indicated that the primary task may require less cerebral processing capacity. The longer reaction time in the secondary task could also suggest a poorer ability to process multiple inputs, a common situation in many sports. The athletes in taekwondo and many other sports have to be able to read and predict opponent’s next move while performing an attack or defensive task. The better performance in the secondary task implies that a greater attention capacity can be allocated to assess opponent’s movements while performing the primary task. This increased ability would provide a great advantage in many sports.

The commonly measured reaction time, the sum of PRT, motor reaction time, and movement time, may be inadequate to distinguish elite and sub-elite athletes or to identify subtle impairments in performance. This is especially true when the required movement is the main action that has been practiced numerous times by the subjects [29]. In addition, the commonly measured reaction time is a combination of central (PRT) and peripheral factors (motor reaction time, movement time), making it difficult to identify the origin of fatigue. In fact, motor reaction time, movement time, and total reaction time were similar after each match in both trials. In addition, total power output during the 3 matches was also unchanged in both trials. These results indicated that the peripheral neuromuscular system was not fatigued during the entire test period. The impairments in premotor reaction time and performance in the secondary task appeared before peripheral fatigue in this group of well-trained taekwondo athletes. By applying the protocols used in this study, we were able to identify the beneficial effect of the combined supplementation on the signal processing stage in the central nervous system, but not at the peripheral neuromuscular level. Similarly, it has been shown that well-trained wrestlers can maintain total power output in 3 simulated matches similar to the protocols used in this study [31].

The standardized diet consumed for two days prior to the trials provided less energy and carbohydrate than the subjects required. It has been suggested that an insufficient carbohydrate supply may impair cognitive function during prolonged exercise [40]. However, our subjects appeared to maintain sufficient muscle glycogen levels as they were euglycemic and plasma lactate concentration and average power output were similar across the 3 simulated matches in both trials. Thus, the effect of hypocaloric diet on the cognitive function in this study may be negligible.

## Conclusions

In conclusion, this study suggested that the combined supplementation of BCAA, arginine, and citrulline could alleviate the exercise-induced central fatigue in elite athletes. By applying validated taekwondo-specific reaction test, the performance in PRT and the secondary task provide more accurate indicator for the processing capacity in the brain. The improvements in these abilities by the supplementation could carry significant benefit in many sports. The effect of this supplementation regime on performance in more complicated skills in various sports warrants further investigation. In addition, it is noteworthy that central fatigue appears before the decline in physical performance in well-trained athletes.

## Abbreviations

BCAA: branched-chain amino acids
CI: confidence interval
EMG: Electromyography
NEFA: non-esterified fatty acids
NO: nitric oxide
PRT: premotor reaction time
